# Optimizing Postoperative Analgesia in Total Knee Arthroplasty: A Randomized Controlled Trial on the Efficacy of Perineural Dexamethasone With iPACK and Adductor Canal Block

**DOI:** 10.1097/AJP.0000000000001344

**Published:** 2025-12-19

**Authors:** Tomasz Reysner, Grzegorz Kowalski, Aleksander Mularski, Anna Perek, Przemysław Daroszewski, Malgorzata Reysner

**Affiliations:** Department of * Palliative Medicine; ‡ Clinical Anesthesiology and Pain Management; § Organization and Management in Health Care, Poznań University of Medical Sciences, Poznań, Poland; † Department of Forensic Medicine, Institute of Medical Sciences Collegium Medicum, University of Zielona Góra, Zielona Góra, Poland

**Keywords:** total knee arthroplasty, dexamethasone, regional anesthesia, opioid-sparing, postoperative pain

## Abstract

**Objectives:**

To assess the effect of perineural dexamethasone on analgesia duration and opioid consumption when used as an adjuvant to the iPACK (infiltration between the popliteal artery and capsule of the knee) block and adductor canal block in patients undergoing total knee arthroplasty.

**Methods:**

In this double-blind, randomized controlled trial, 60 patients aged 65 years or older undergoing total knee arthroplasty under spinal anesthesia were assigned to one of 2 groups. The control group received iPACK and adductor canal block with 0.2% ropivacaine alone, while the Dexamethasone group received the same blocks with the addition of perineural dexamethasone. The primary outcome was time to first opioid rescue analgesia. Secondary outcomes included total opioid consumption over 48 hours, postoperative pain scores at defined time points, quadriceps muscle strength, and adverse effects, including neurological complications and hyperglycemia.

**Results:**

Patients receiving dexamethasone experienced a significantly longer duration of analgesia (15.9±1.2 h vs. 8.8±1.6 h), lower total opioid consumption over 48 hours (1.2±1.3 mg vs. 2.3±1.4 mg morphine equivalents), and fewer patients required opioids (20% vs. 50%). Pain scores were significantly lower at 8 and 12 hours postoperatively. No differences were observed in motor function or adverse event rates.

**Discussion:**

Perineural dexamethasone enhanced the duration and quality of postoperative analgesia without compromising motor function. Its inclusion in regional analgesia protocols for total knee arthroplasty may contribute to improved recovery and reduced opioid use.

Total knee arthroplasty (TKA) is a widely performed surgical procedure to alleviate pain and improve joint function in patients with end-stage knee osteoarthritis.^1^ However, postoperative pain remains a significant challenge, particularly in geriatric patients,^2^ as inadequate pain control can hinder early mobilization, prolong hospital stays, and increase opioid consumption; thereby elevating the risk of opioid-related adverse effects such as respiratory depression, nausea, vomiting, and cognitive impairment.^3^ Consequently, there is an increasing focus on multimodal analgesia strategies to enhance postoperative pain relief while minimizing opioid use.^4^

Regional anesthesia techniques have gained prominence in TKA because they provide effective pain relief while preserving motor function.^5^ The adductor canal block (ACB) and infiltration between the popliteal artery and capsule of the knee (iPACK) block are 2 well-established nerve blocks that selectively target sensory pathways, reducing postoperative pain without significantly impairing quadriceps muscle strength.^6,7^ The ACB primarily anesthetizes the saphenous nerve and the nerve to the vastus medialis.^8^ In contrast, the iPACK block targets the posterior knee capsule, a key pain generator in TKA.^9^ When used together, these techniques offer comprehensive analgesia, allowing for early ambulation and functional recovery.^10^

A growing body of evidence suggests that adding perineural adjuvants to local anesthetics can further enhance the duration and efficacy of nerve blocks. Among these adjuvants, dexamethasone (DEX) has gained attention due to its potent anti-inflammatory properties and ability to prolong the effects of local anesthetics.^11^ Studies have shown that perineural DEX can significantly extend analgesia duration and reduce opioid requirements in various peripheral nerve blocks.^12^ The mechanism by which DEX exerts these effects is multifaceted, suppressing neurogenic inflammation, inhibiting nociceptive signaling, and enhancing sodium channel blockade by local anesthetics.^13^

Despite its potential benefits, the efficacy and safety of perineural DEX as an adjuvant to the combined iPACK and ACB blocks in TKA remain underexplored, particularly in geriatric populations who are at a higher risk of opioid-related complications. The present study aims to address this gap by evaluating the impact of perineural DEX on postoperative pain relief, opioid consumption, and functional recovery following TKA in patients aged 65 years and older.

We hypothesize that the addition of perineural DEX to iPACK and ACB will prolong the time to first opioid administration, reduce total opioid consumption, and enhance early postoperative pain control without compromising motor function. Unlike previous studies such as Zeng et al,^11^ which included mixed-age cohorts and did not assess motor function or glycemic safety, our study focuses exclusively on a geriatric population (≥65 y), introduces routine quadriceps strength assessment, and monitors postoperative blood glucose levels. We also used a standardized spinal anesthesia protocol with mild sedation specifically designed for older adults, ensuring both internal validity and relevance to high-risk surgical populations. Given the increasing emphasis on opioid-sparing pain management strategies, findings from this study could have significant implications for optimizing regional anesthesia techniques in TKA.

This randomized controlled trial (RCT) compared 2 groups: one receiving the standard iPACK and ACB with ropivacaine alone and the other receiving iPACK and ACB with ropivacaine and perineural dexamethasone. Key outcomes included time first to rescue opioid analgesia, total opioid consumption over 48 hours, postoperative pain scores, quadriceps muscle strength, and potential adverse effects such as nerve damage and hyperglycemia.

By rigorously evaluating these parameters, our study sought to contribute valuable clinical insights into the role of perineural DEX in multimodal analgesia protocols for TKA, ultimately facilitating improved pain management and enhanced recovery pathways for geriatric patients.

## METHODS

### Study Design

This double-blinded, prospective, RCT was conducted at a single orthopedic center in Poland. The Poznan University of Medical Sciences Bioethics Committee approved the study protocol on September 13, 2023 (protocol number 541/2023). Subsequently, the trial was registered at ClinicalTrials.gov (NCT06470204) on August 13, 2024. Before participation, written informed consent was obtained from all patients following ethical research standards. Patient enrollment occurred between August 14, 2024, and January 31, 2025. The study was conducted in full compliance with the principles outlined in the Declaration of Helsinki.

### Participants

Patients scheduled for TKA under spinal anesthesia were considered for enrollment before surgery. Inclusion criteria encompassed individuals aged 65 to 100 years who could provide informed consent and reliably report symptoms to the research team.

Exclusion criteria included patients who declined participation or could not provide first-party consent due to cognitive impairment or language barriers.

In addition, patients with failed spinal anesthesia were excluded because the iPACK and ACB block protocol was designed specifically as an adjunct to neuraxial anesthesia. Conversion to general anesthesia would introduce analgesic and pharmacological confounders, thereby compromising the internal validity of group comparisons.

### Randomization and Blinding Procedures

A computer-generated randomization process assigned patients in a 1:1 ratio to one of the following groups: the Control group received an ultrasound-guided iPACK block and ACB with 0.2% ropivacaine, and the DEX group received an ultrasound-guided iPACK block and ACB with 0.2% ropivacaine and perineural DEX. The randomization list was generated using the nQuery Advisor program (Statistical Solutions, Boston, MA) by a researcher who was not otherwise involved in the study. Before the orthopedic procedure, the “first” consultant anesthesiologist received and opened the assigned envelope and administered the iPACK block and ACB, or iPACK block and ACB with perineural DEX according to the allocated group. Immediately after performing the nerve blocks, the “first” anesthesiologist was replaced by a “second” consultant anesthesiologist, who remained blinded to the group assignment and supervised all anesthesia-related procedures during surgery. This was done to preserve strict double-blinding, not only for postoperative outcome assessors but also to blind intraoperative anesthesia management. This eliminated potential bias in fluid administration, sedation titration, or anesthetic adjustments that might be unconsciously influenced by knowledge of group allocation. If any intraoperative complication (eg, unexpected conversion to bilateral surgery, medical instability requiring ICU care) occurred after administration of the nerve block but before protocol completion, the patient was classified as having discontinued intervention due to surgical complication.

Throughout the study, the anesthesia team, surgical team, operating room staff, patients, and outcome assessors remained blinded to the intervention received. Group allocation was only revealed after the completion of statistical analyses to prevent bias.

### Procedures

In both study groups, patients received an individualized midazolam dose (2.5 to 7.5 mg p.o.) approximately 30 minutes before surgery as part of a multimodal preemptive analgesia protocol. The dose was tailored based on age, weight, frailty index, and comorbidities to avoid oversedation in elderly patients. Most patients received 2.5 to 5.0 mg; 7.5 mg was reserved only for individuals with documented benzodiazepine tolerance.

Spinal anesthesia was performed first, followed by the nerve blocks, to enhance patient comfort and minimize movement during block placement.

Sedation during spinal and block procedures was achieved using a carefully titrated propofol infusion (typically 1 to 2.5 mg/kg/h in this elderly population, with a maximum limit of 5 mg/kg/h only in rare cases). This approach was chosen to maintain light sedation (Modified Observer’s Assessment of Alertness/Sedation Scale score of 3 to 4) with preserved spontaneous ventilation.

Propofol infusion rates were adjusted individually, and all patients were continuously monitored with capnography, pulse oximetry, and ECG to ensure safety. Oxygen was administered via facemask at 3 to 5 L/min depending on respiratory needs.

Spinal anesthesia was performed at the L3/4 interspace using a 27 G, 90 mm Sprotte needle (PAJUNK) with 4 mL of 0.5% ropivacaine. The surgeon performed no periarticular infiltration during the procedure. The “first” and “second” anesthesiologists involved in this study had over 5 years of postspecialty clinical experience focusing on regional anesthesia, specifically nerve blocks.

### iPACK and Adductor Canal Block (ACB) Procedures

All nerve blocks were performed under ultrasound guidance following the administration of spinal anesthesia.

For the **iPACK block**, a low-frequency curvilinear transducer was used to identify the interfascial plane between the femur and the popliteal artery. Using an in-plane approach, 20 mL of 0.2% ropivacaine was injected posterior to the artery. In the DEX group, 4 mg (1 mL) of DEX was added perineurally. Needle placement and spread were confirmed by hydro-dissection and real-time ultrasound imaging.

For the **ACB**, a high-frequency linear transducer was placed at mid-thigh to visualize the femoral artery and surrounding structures. A 22-gauge needle was advanced under ultrasound guidance into the adductor canal. Twenty milliliters of 0.5% ropivacaine was injected, with or without 4 mg dexamethasone, depending on group assignment. Needle location and injectate distribution were confirmed using hydro-dissection.

Both blocks were performed by experienced anesthesiologists, with careful aspiration to avoid intravascular injection. Block duration did not exceed 5 minutes per technique.

### Surgical and Postoperative Protocol

All patients underwent knee arthroplasty under spinal anesthesia by a single surgical team of 4 orthopedic surgeons at the Orthopedic Hospital, Poznan University of Medical Sciences. The Direct Superior Approach (DSA) was consistently used across all patients, and an uncemented implant (Smith & Nephew Polarstem/R3) was implanted in each case.

### Surgical Protocol for Total Knee Arthroplasty (TKA)

All patients underwent TKA) under spinal anesthesia, performed by a single surgical team of 4 experienced orthopedic surgeons at the Orthopedic Hospital, Poznan University of Medical Sciences. The medial parapatellar approach was consistently utilized across all patients to ensure surgical standardization. A cemented, posterior-stabilized total knee prosthesis was implanted in all cases, with bone preparation and soft tissue balancing performed according to standard TKA protocols. A tourniquet was applied before skin incision and was inflated throughout the procedure to minimize intraoperative blood loss. Patellar resurfacing was performed selectively based on preoperative radiographic and intraoperative findings. Following prosthesis implantation and final component fixation, thorough wound irrigation was performed, and a suction drain was placed at the surgeon’s discretion. The joint capsule and deep soft tissues were closed in layers, and skin closure was performed using subcuticular sutures or skin staples.

### Postoperative Protocol

In the postoperative period, all patients followed an enhanced recovery after surgery (ERAS) protocol, including multimodal analgesia, early mobilization, and venous thromboembolism (VTE) prophylaxis. Weight-bearing was encouraged on the first postoperative day, and all patients underwent structured physiotherapy with supervised rehabilitation sessions. Postoperative pain management followed a multimodal analgesic protocol, incorporating a combination of nonopioid and opioid analgesics to optimize pain control while minimizing opioid consumption. The regimen included Acetaminophen 1.0 g every 6 hours, Metamizole 1.0 g every 6 hours, and Ibuprofen 400 mg every 8 hours. For rescue analgesia, if a patient’s Numerical Rating Scale (NRS) score reached ≥4, a 5 mg intravenous oxycodone bolus was administered. To ensure standardized reporting and facilitate comparisons with other studies, total opioid consumption was expressed in morphine milliequivalents (mEQ), using a standard conversion ratio of 1 mg oxycodone=1.5 mg morphine.

All patients received daily enoxaparin for 4 weeks postoperatively as prophylaxis against thromboembolic events. Early mobilization was initiated 10 hours after surgery, with patients ambulating under supervision using a hiker to promote functional recovery and reduce postoperative complications.

All patients were monitored for postoperative complications, including infection, deep vein thrombosis, and range-of-motion limitations.

### Outcome Measures

The *primary outcome* was the time to the first administration of rescue opioid analgesia, which was assessed by residents from the postoperative and orthopedic wards who were not involved in the study.

*Secondary outcomes* included total opioid consumption, which was recorded in milligrams of oxycodone from the orthopedic ward records. This data was converted into morphine milliequivalents (mEQ) using the standardized conversion ratio (1 mg oxycodone=1.5 mg morphine) to ensure consistency with other studies. Residents and fellows who were blinded to the study performed these assessments. Pain intensity was evaluated using the Numerical Rating Scale (NRS) at predefined postoperative time points (4, 6, 12, and 24 h after surgery). All NRS scores were assessed at rest to ensure consistency and reproducibility across patients. The NRS ranged from 0 (no pain) to 10 (worst pain imaginable). Two independent physicians conducted these evaluations, and the final pain score was determined through mutual agreement at the end of the assessment. Quadriceps muscle strength was assessed using the Medical Research Council (MRC) Scale for Muscle Strength, where Grade 5 represents normal muscle strength, grade 4 indicates movement against gravity and resistance, Grade 3 indicates movement against gravity over nearly the entire range, grade 2 indicates movement of the limb but not against gravity, Grade 1 represents visible contraction without movement of the limb (not applicable to hip flexion), and Grade 0 indicates no visible contraction. Two independent physicians evaluated quadriceps strength, and the final score was determined by consensus at the end of the assessment.

Nerve injury was assessed retrospectively on the day of discharge based on documented neurological deficits in the orthopedic ward records. Nerve deficits were classified as follows: 0: No nerve damage, 1: Minor sensory paresthesia, 2: Complete sensory anesthesia, 3: Complete motor deficit with or without sensory paresthesia, 4: Complex Regional Pain Syndrome.^12^ Two researchers, blinded to group allocation, reviewed and classified these outcomes.

Blood glucose levels were measured at 12, 24, and 48 hours postoperatively. Blood samples were collected by nursing staff, blinded to the study, and analyzed by 2 independent researchers who were also blinded to group allocation.

### Statistical Analysis

The sample size was determined based on our central hypothesis, which stated that the duration until the first rescue opioid analgesia would be significantly longer in the DEX group compared with the control group. Conversely, our null hypothesis asserted that there would be no significant difference in the time until the first rescue analgesia between these 2 groups. The time to first rescue opioid analgesia served as the primary variable. From a pilot study involving 10 patients who were not included in the final analysis, we found that the time to first rescue opioid analgesia was 8.46±4.52 hours (mean±SD) for the control group and 14.58±4.29 hours (mean±SD) for the DEX group. Through pairwise comparison, we estimated that a sample size of 44 patients is necessary to detect a difference in the time to first rescue opioid analgesia between the 2 groups, ensuring a statistical power of 95% at a *P*-value of <0.05. To facilitate block randomization and account for potential loss to follow-up, we planned for 30 patients in each group, totaling 60 patients recruited. Statistical analysis was performed using GraphPad Prism 10.1.1 (270) software (GraphPad Software Inc., San Diego, CA). The parametric distribution of numerical variables was evaluated using the Shapiro-Wilk normality test. Differences between groups were assessed using the *t* test or Mann-Whitney *U* test. Categorical variables were compared with Fisher exact test conducted contingency analysis between groups. In addition, the Wilcoxon signed-rank test was used for paired within-group comparisons to assess changes in pain scores over time. A *P*-value of <0.05 was considered statistically significant and was calculated with 95% Cl.

## RESULTS

**FIGURE 1:**
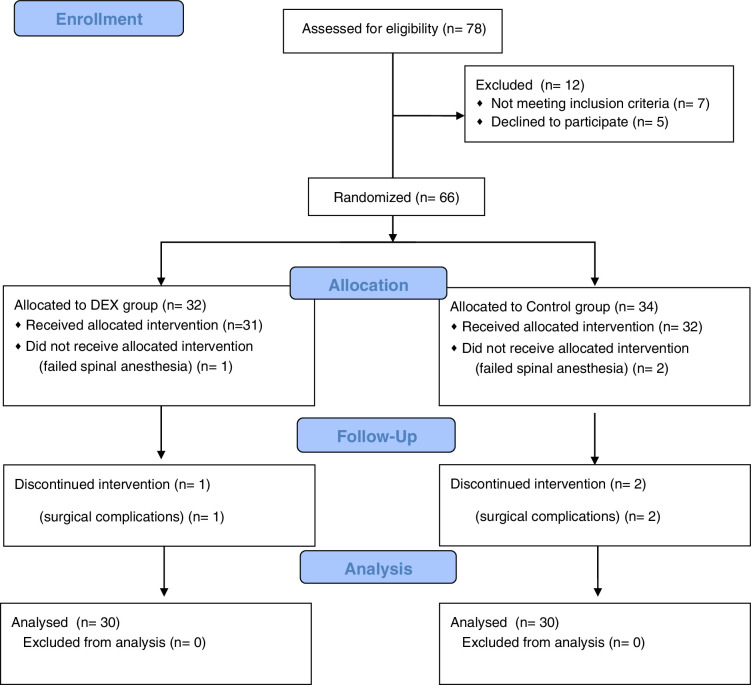
Consort-2010-flow-diagram.

Seventy-eight patients were assessed for eligibility. Twelve were excluded: 7 did not meet the inclusion criteria and 5 declined to participate. Sixty-six patients were randomized into 2 groups. In the DEX group, 32 patients were allocated to receive the intervention. One patient did not receive the intervention due to failed spinal anesthesia, and 31 received the allocated treatment. One of those later discontinued the intervention due to surgical complications. In total, 30 patients were included in the final analysis, with no exclusions (*see Supplementary Table 1*, Supplemental Digital Content 1, http://links.lww.com/CJP/B270). In the control group, 34 patients were allocated. Two patients did not receive the intervention due to failed spinal anesthesia, and 32 proceeded with the assigned treatment. Two of those discontinued the intervention due to surgical complications. Thus, 30 patients were also included in the final analysis (Fig. 1). The study maintained a high retention rate, ensuring adequate statistical power. Baseline characteristics were comparable between the groups (Table 1).

**TABLE 1:** Baseline Characteristics

	Control group (n=30)	DEX group (n=30)	*P*
ASA			
ASA II	10	9	>0.9999
ASA III	20	21	
Co-morbideties			0.6354
Diabetes mellitus	12	17	
Hypertension	25	28	
Heart disease	12	10	
Age (y)	71.3±3.5 (66-78)	72.0±2.9 (67-78)	0.4282
M/F	12/18	16/18	
BMI	29.6±2.8 (25.0-36.0)	30.2±2.7 (26.0-37.0)	0.4086
Duration of surgery (min)	79.0±6.7 (65-95)	77.3±7.3 (65-90)	0.4989

*P*-value compares the control group to the DEX group.

ACB indicates adductor canal block; BMI, mody mass index; DEX, dexamethasone; F, female; iPACK infiltration between the popliteal artery and capsule of the knee block; M, male; min, minutes.

### Primary Outcome

The time to first rescue opioid analgesia was significantly prolonged in the DEX group (15.9±1.2 h) compared with the control group (8.8±1.6 h) (*P <* 0.0001). The mean difference was 7.8 hours (95% CI: 6.5 to 8.0), indicating a substantial analgesia extension with dexamethasone.

### Secondary Outcomes

**FIGURE 2:**
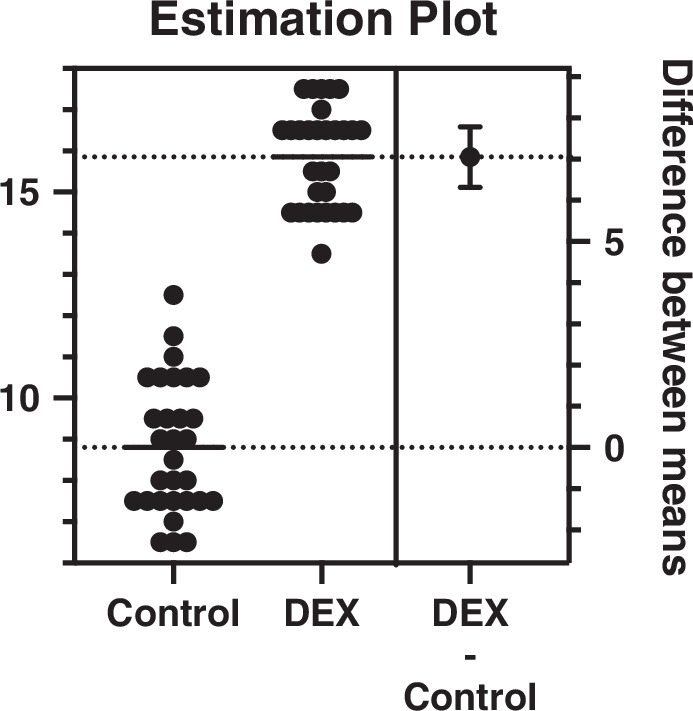
Time to first rescue opioid analgesia.

Forty-eight-hour opioid consumption was significantly lower in the DEX group (1.2±1.3 morphine mEQ) compared with the control group (2.3±1.4 morphine mEQ) (*P=*0.0009). The mean difference was −1.5 morphine mEQ (95% CI: −2.0 to 0.0), suggesting an opioid-sparing effect with dexamethasone, as seen in Table 2 and Figure 2.

**TABLE 2:** Primary and Secondary Outcomes

	Control group (n=30)	DEX group (n=30)	*P*	Mean difference (95% Cl)
Time to first rescue opioid analgesia (h)	8.8±1.6	15.9±1.2	**<0.0001**	7.8 (6.5 to 8.0)
48 h opioid consumption (morphine mEQ)	2.3±1.4	1.2±1.3	**0.0009**	−1.5 (−2.0 to 0.0)
Postoperative opioid consumption				
Yes	24	**15**	0.0292	NA
** **No	6	15		
NRS				
** **4 h	1.5±0.6	1.4±1.7	0.8653	NA
8 h	2.4±0.7	1.2±0.9	**<0.0001**	−0.5 (−2.0 to −1.0)
12 h	2.6±0.5	1.6±0.5	**<0.0001**	−1.0 (−1.0 to −1.0)
24 h	2.3±0.5	2.3±0.6	>0.9999	NA
48 h	2.5±0.5	2.3±0.5	0.0833	NA
Quadriceps muscle strength				
Knee extension				
4 h	5.0 (0)	5.0 (0)	>0.9999	NA
8 h	5.0 (0)	5.0 (0)	>0.9999	NA
12 h	5.0 (0)	5.0 (0)	>0.9999	NA
24 h	5.0 (0)	5.0 (0)	>0.9999	NA
Hip adduction				
4 h	5.0 (0)	5.0 (0)	>0.9999	NA
8 h	5.0 (0)	5.0 (0)	>0.9999	NA
12 h	5.0 (0)	5.0 (0)	>0.9999	NA
24 h	5.0 (0)	5.0 (0)	>0.9999	NA
Nerve damage (0-4)				
	0 (0)	0 (0)	>0.9999	NA
Blood glucose				
12h	108±11.2	107.7±13.8	0.9346	NA
24h	118.7±21.33	111.8±18.0	0.1980	NA
48h	111.6±12.7	112.1±16.3f	0.8284	NA

Statistically significant differences are marked in bold.

*P*-value compares the control group to the DEX group.

Values are presented as mean ± SD or count.

Statistical significance was determined using *t* test, Mann-whitney, or Fisher exact test where appropriate.

ACB, indicates adductor canal block; DEX, dexamethasone; h, hours; iPACK infiltration between the popliteal artery and capsule of the knee block mEQ, milliequivalents; NA, not applicable.

A significantly lower proportion of patients in the DEX group (15 out of 30, 50%) required postoperative opioid consumption compared with the control group (24 out of 30, 80%) (*P=*0.0292), further supporting the opioid-sparing benefits of dexamethasone.

Pain scores, recorded at rest, at 8 and 12 hours postoperatively were significantly lower in the DEX group compared with the control group (*P <*0.0001 for both time points). No significant differences in pain scores were observed at 4 hours (*P*=0.8653), 24 hours (*P* >0.9999), or 48 hours (*P*=0.0833).

Quadriceps muscle strength, assessed through knee extension and hip adduction at 4, 8, 12, and 24 hours postoperatively, remained identical in both groups (mean score: 5.0 at all time points, *P* >0.9999), indicating that dexamethasone did not impair muscle function.

Nerve damage was not observed in either group, as all patients scored 0 (*P* >0.9999).

Blood glucose levels at 12, 24, and 48 hours did not differ significantly between groups (*P=*0.9346*, P=*0.1980, and *P=*0.8284, respectively), indicating that dexamethasone did not induce hyperglycemia within the monitored timeframe.

## DISCUSSION

The findings of our study provide compelling evidence supporting the efficacy of perineural DEX as an adjuvant to iPACK and adductor canal block (ACB) in TKA. Our results demonstrate that the addition of perineural dexamethasone significantly prolongs the duration of analgesia, reduces postoperative opioid consumption, and enhances early postoperative pain control without compromising motor function. These findings align with previous literature suggesting that corticosteroid adjuvants can enhance regional anesthesia outcomes by prolonging the duration of nerve blockade and reducing systemic opioid use.^14,15^

### Prolonged Analgesia Duration With Perineural Dexamethasone

Our study found that the DEX group had a significantly longer time to first opioid rescue analgesia than the control group. This result is consistent with previous studies evaluating the role of DEX as a perineural adjuvant in peripheral nerve blocks.^12,16,17^ DEX is believed to exert analgesic effects by inhibiting neurogenic inflammation, attenuating ectopic discharges in injured nerves, and enhancing sodium channel blockade when co-administered with local anesthetics.^18,19^ These mechanisms collectively contribute to significantly prolonging analgesic duration.^18^ Few studies have investigated the role of perineural DEX in TKA. Li et al^20^ found that DEX significantly extended the analgesia duration in TKA patients when added to femoral and sciatic nerve block. Similarly, a retrospective cohort study by Shoni et al^6^ concluded that perineural DEX could prolong the duration of nerve blocks by up to 12 hours, supporting our findings that DEX enhanced analgesia in the DEX group.

### Reduction in Postoperative Opioid Consumption

Another key finding of our study was the significant reduction in total opioid consumption in the DEX group compared with the control group. Notably, 50% of patients in the DEX group avoided opioid use altogether compared with only 20% in the control group. These findings are clinically significant, given the ongoing opioid crisis and the need for opioid-sparing analgesia strategies in postoperative care.^21^ Our results align with Del Toro-Pagán et al,^22^ who demonstrated that perineural DEX significantly reduces opioid requirements in patients undergoing lower limb surgery. Moreover, Zeng et al^11^ reported that combining dexamethasone with adductor canal and iPACK blocks decreased opioid consumption by approximately 40% in TKA patients. The opioid-sparing effect of DEX is particularly beneficial in geriatric patients,^23^ who are at higher risk of opioid-related side effects such as delirium, respiratory depression, and gastrointestinal complications.^24,25^

### Improved Early Postoperative Pain Control

Pain scores at 8 and 12 hours postoperatively were significantly lower in the DEX group compared with the control group. However, there were no significant differences in pain scores at 4 hours, 24 hours, or 48 hours. These findings suggest that DEX primarily enhances early postoperative pain relief, which is critical in facilitating early mobilization and rehabilitation following TKA. A study by Li et al^20^ similarly found that patients receiving DEX as an adjuvant to regional anesthesia had significantly lower pain scores at 6 to 12 hours postoperatively. In addition, Akaravinek et al^26^ emphasized that prolonging regional anesthesia duration with dexamethasone improves functional recovery by enabling earlier weight-bearing and ambulation in TKA patients.

### Preservation of Quadriceps Muscle Function

A significant concern with perineural adjuvants is the potential for motor impairment, which could hinder early rehabilitation efforts in TKA patients. In our study, quadriceps muscle strength remained unaffected between the DEX and control groups. These results confirm that perineural DEX does not negatively impact motor function, allowing patients to participate in rehabilitation protocols fully.^27^

Our findings are consistent with those of Zeng et al,^11^ who reported that DEX does not compromise motor function when used as an adjuvant to sensory nerve blocks. In addition, Shoni et al^6^ found that DEX primarily prolongs sensory block duration without significantly affecting motor nerves, making it an ideal adjuvant for motor-sparing regional anesthesia techniques like the ACB and iPACK blocks.

### Absence of Significant Adverse Effects

Our study found no significant differences in nerve damage or blood glucose levels between the DEX and control groups at all time points. This suggests that perineural DEX does not increase the risk of nerve injury or induce hyperglycemia within the observed timeframe.

Concerns regarding potential neurotoxicity with perineural DEX administration have mainly been unfounded in clinical studies. Reysner et al^12^ reported no evidence of nerve injury in children receiving perineural DEX, further supporting the safety profile of DEX in regional anesthesia. Moreover, while corticosteroids have the potential to cause transient hyperglycemia, our study and others^11^ suggest that a single-dose perineural administration does not result in clinically significant blood glucose alterations.

### Clinical Implications

Our findings have significant clinical implications for enhancing multimodal analgesia protocols in TKA. The use of perineural DEX in combination with iPACK and ACB provides a safe and effective method to prolong analgesia, reduce opioid consumption, and improve early pain control.^5^ Distinct from Zeng et al,^11^ our study focuses on a geriatric population—an understudied and vulnerable cohort with a heightened risk of opioid-related complications. We uniquely incorporated structured quadriceps strength assessments to verify motor-sparing efficacy and implemented glucose monitoring to evaluate systemic corticosteroid safety. The anesthesia approach, based on spinal block under tailored sedation, mirrors real-world clinical practice for older patients and adds to the external validity of our findings.^28^ Moreover, full blinding of anesthesiologists, surgical staff, and outcome assessors minimizes performance and detection bias, strengthening the rigor of our RCT.

Given the rising prevalence of TKA in the geriatric population, optimizing regional anesthesia strategies to minimize opioid use is of paramount importance. The findings of our study reinforce recommendations by the American Society of Regional Anesthesia and Pain Medicine (ASRA), which advocate for opioid-sparing techniques in perioperative pain management. Furthermore, our study supports the integration of dexamethasone into multimodal pain management protocols, aligning with the Enhanced Recovery After Surgery (ERAS) guidelines for TKA.

### Limitations and Future Directions

While our study demonstrates the benefits of perineural DEX in TKA, it has limitations. The single-center design may limit generalizability, and the short follow-up period (48 h) does not assess long-term pain or functional recovery. In addition, interindividual variability in pain sensitivity and opioid use may have influenced outcomes. Moreover, patients requiring conversion to general anesthesia due to failed spinal block were excluded, as this would have introduced confounding variables that conflict with the standardized neuraxial protocol used for all study participants.

Future research should focus on determining the optimal DEX dosage to maximize analgesia while minimizing systemic effects. Longer follow-up studies are needed to evaluate its impact on chronic postsurgical pain and functional recovery. Investigating alternative corticosteroids, such as methylprednisolone, may help identify superior efficacy or safety profiles. Addressing these gaps will refine multimodal analgesia strategies, improving pain management and recovery in TKA patients.

## CONCLUSIONS

In conclusion, our study demonstrates that perineural DEX significantly enhances the analgesic efficacy of iPACK and adductor canal blocks in TKA patients, leading to prolonged analgesia, reduced opioid consumption, and improved early pain control without impairing motor function or increasing adverse effects. These findings support the routine use of perineural DEX as an effective adjuvant for optimizing multimodal analgesia in TKA.

## Supplementary Material
