# Supplementary material for: Optimizing Postoperative Analgesia in Total Knee Arthroplasty: A Randomized Controlled Trial on the Efficacy of Perineural Dexamethasone With iPACK and Adductor Canal Block
Source: Clin J Pain. 2025 Dec 19;42(2):e1344. doi: 10.1097/AJP.0000000000001344 (PMC12788753; doi:10.1097/AJP.0000000000001344)
Supplement: Supplementary file 1 [file ajp-42-e1344-s001.docx]

# Supplementary Table 1. Study Exclusions by Group

| Group | Reason for Exclusion | Number of Patients |
| --- | --- | --- |
| Control | Failed spinal anesthesia | 2 |
| Control | Surgical complication after block | 2 |
| DEX | Failed spinal anesthesia | 1 |
| DEX | Surgical complication after block | 1 |
| Total Excluded | — | 6 |

*Note: Surgical complications occurred after block placement and prior to complete outcome assessment, leading to discontinuation from protocol.*
